# Use of somatic mutations to quantify random contributions to mouse development

**DOI:** 10.1186/1471-2164-14-39

**Published:** 2013-01-18

**Authors:** Wenyu Zhou, Yunbing Tan, Donovan J Anderson, Eva M Crist, Hannele Ruohola-Baker, Stephen J Salipante, Marshall S Horwitz

**Affiliations:** 1 Department of Biology, University of Washington, Seattle, WA, 98195, USA; 2 The School of Electrical Engineering and Computer Science, Washington State University, Pullman, WA, 99163, USA; 3 Department of Pathology, University of Washington, Box 358056, Seattle, WA, 98195, USA; 4 Department of Biochemistry, University of Washington, Seattle, WA, 98195, USA; 5 Department of Laboratory Medicine, University of Washington, Seattle, WA, 98195, USA

**Keywords:** Fate map, Cell lineage, Differentiation

## Abstract

**Background:**

The *C. elegans* cell fate map, in which the lineage of its approximately 1000 cells is visibly charted beginning from the zygote, represents a developmental biology milestone. Nematode development is invariant from one specimen to the next, whereas in mammals, aspects of development are probabilistic, and development exhibits variation between even genetically identical individuals. Consequently, a single defined cell fate map applicable to all individuals cannot exist.

**Results:**

To determine the extent to which patterns of cell lineage are conserved between different mice, we have employed the recently developed method of “phylogenetic fate mapping” to compare cell fate maps in siblings. In this approach, somatic mutations arising in individual cells are used to retrospectively deduce lineage relationships through phylogenetic and—as newly investigated here—related analytical approaches based on genetic distance. We have cataloged genomic mutations at an average of 110 mutation-prone polyguanine (polyG) tracts for about 100 cells clonally isolated from various corresponding tissues of each of two littermates of a hypermutable mouse strain.

**Conclusions:**

We find that during mouse development, muscle and fat arise from a mixed progenitor cell pool in the germ layer, but, contrastingly, vascular endothelium in brain derives from a smaller source of progenitor cells. Additionally, formation of tissue primordia is marked by establishment of left and right lateral compartments, with restricted cell migration between divisions. We quantitatively demonstrate that development represents a combination of stochastic and deterministic events, offering insight into how chance influences normal development and may give rise to birth defects.

## Background

Mouse gestation takes approximately 20 days [1], and, although cell cycle length is variable, embryonic cells divide about twice per day [2]. It can therefore be surmised that about 40 or so mitotic generations transpire between fertilization and birth—a value similar to other estimates derived from different assumptions [3]. If all embryonic cell divisions produced two daughter cells that both subsequently divided, then a newborn mouse should be composed of 2^40^ (≈10^11^) cells. Given that the mass of a cell is about 10^-12^ kg [4], a newborn mouse would weigh about 10 g—close to actual measurements nearer to just 1 g [1]. However, each of the two daughter cells may experience different fates; both daughter cells do not always divide, nor do they do so at the same time. Along with the effects of apoptosis, this accounts for the fact that a newborn mouse has fewer cells than anticipated if embryonic cell proliferation were to proceed exponentially.

In fact, asymmetric cell divisions are evident in the *C. elegans*‘ cell fate map, in which the lineage of every cell in the worm, beginning from the zygote, is charted [5]. Based on the cell fate map, it becomes apparent that sometimes one daughter cell continues to proliferate while the other ceases to divide and undergoes terminal differentiation or death. There are then only two types of proliferative cell divisions, distinguishable by how they are graphed on the lineage tree: one type in which both daughter cells divide and the other where only one daughter cell continues to divide. If only the first of these two possibilities were to hold constant—that daughter cells constitutively divide—then there would only be one possible cell lineage tree, a symmetric one with each node bifurcating at every branch. However, the addition of the second possible type of cell division—in which one of the two daughter cells ceases to further divide—adds significant complexity to the repertoire of potential cell lineage trees and consequently to the different types of tissue and body plans that can be created during embryogenesis.

For any given number of *n* cells in an embryo there are a surprisingly large possible number ((2*n*-3)!/2^*n*-2^(*n*-2)!)) of potential cell lineage histories [6]. For an embryo with 4 cells there are 15 different possible fate maps, for 8 cells there are 135,135, and for 16 cells the number exceeds 10^15^. For the thousand or so cells of the adult worm [5], the number of potential different lineage histories is immeasurably large. Yet, all individual worms invariantly develop identically; the cell fate map remains constant from one *C. elegans* specimen to the next [5].

For many animals, however, including mice and other mammals, there does not exist a single, defined fate map in which the same developmental plan is followed by all individuals of that species. Instead, development is partly stochastic [7]. In contrast to *C. elegans*, any given cell from an early embryo is totipotent and can adopt any of a number of different possible cell fates. Commitment to any particular lineage is probabilistic (as reviewed [6]). A striking illustration of the variable development occurring between even genetically identical individuals of the same species is evident in cloned animals, where size, blood cell indices and serum markers, skin type, hair growth patterns, blood vessel branching and even the number of teats all show considerable heterogeneity, even among constitutionally genetically identical individuals [8]. Similar examples include variable heart valve morphology [9], craniofacial structure [10], and numbers of neurons [11,12] and cortical brain patterning [12] among isogenic strains of rodents. These studies indicate that while genetic background and environment contribute to variation, at least some differences are not genetically determined but are rather inescapable consequences of developmental noise.

Here we attempt to measure the extent to which random versus deterministic factors shape development. We employ an approach that we have dubbed “phylogenetic fate mapping”, previously developed by our group [13-16] and similar to methods developed by others [3,17-21], in which cell lineage histories are inferred from somatic mutations. We have dissected single cells from analogous tissues of two mouse littermates, expanded the cells clonally *ex vivo* in order to obtain sufficient quantities of DNA to perform mutational analysis, cataloged length-altering mutations at dozens of polyguanine (polyG) repeat mutational hotspots dispersed throughout the genome, and determined the order in which mutations have arisen, toward the goal of reconstructing cellular lineages. For the purpose of maximally extracting somatic genetic information, we have additionally introduced a technical refinement in which studies are conducted in DNA repair-deficient hypermutable mouse strains and have also evaluated new methods of inferring cellular ancestry based on genetic distance, in addition to those based on phylogenetics.

## Results

### Mutation profiles of single cells

We have previously carried out phylogenetic fate mapping studies utilizing the developmentally normal “Immortomouse” strain, which expresses a conditional SV40 T-antigen oncogene and conveniently allows for derivation of conditionally-immortalized cell lines [14,22] from clonally expanded single cells. To obtain larger numbers of informative mutations, we took the additional step of breeding the Immortomouse’s conditional T-antigen into hypermutable strains, deficient both in the lagging-strand DNA polymerase delta proof-reading [23,24] and MLH1 DNA mismatch repair [25] activities.

We successfully isolated and cultured as conditionally immortalized clonal cell lines about 100 single cells dissected from various tissues at similar locations from each of two adolescent (5 week) female mouse littermates (here identified as “mouse 1” and “mouse 2”). We harvested cells representing vascular endothelial tissue from the brain, preadipocytes from abdominal fat, and fibroblasts from hindlimb muscles (Additional file 1: Table S1). In addition to mutations developing somatically during the lifetime of the mouse, mutations can also arise during *ex vivo* clonal expansion; however, they are expected to randomly populate only a few cells per clone and because they are unique to each isolate are unlikely to confound inferences of lineage, even if they are detectable. We therefore assume that the most frequent alleles in a clone represent genotypes of the original single cell from which the clone is derived [14,15]. As an additional measure to control for mutations arising during *ex vivo* clonal expansion, for several isolates, we split each clone after just a few passages into two separate cultures and independently genotyped and analyzed each member of the pair to insure that separately they produced equivalent results (see below).

**Figure 1:**
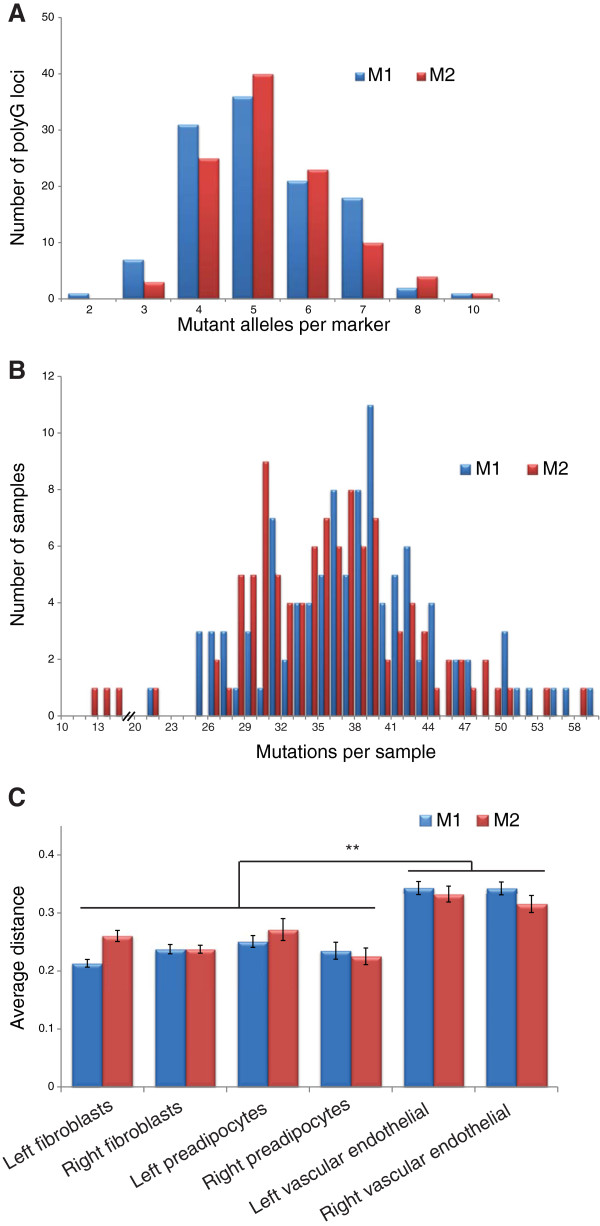
**Somatic polyG mutation profiles of two mouse littermates.** (**A**) Histogram showing how many different mutant alleles are identified for each marker across all of the approximately 100 cells genotyped for each mouse. An average of 5 mutant alleles was observed on each polyG marker in both mice, yielding an average mutation rate as 0.5 mutant alleles/polyG locus/cell. (**B**) Histogram showing the number of polyG marker mutations (from all approximately 110 markers) detected per cell for each mouse. For mouse 1, somatic mutations were observed in 36.7% of approximately 110 polyG markers for each single cell clone on average, while in mouse 2, an average of 34.4% were observed. (**C**) Average genetic distance of different types of tissues to the zygote for each mouse.

To ascertain somatically-acquired mutations in each of the single cell clones, we extracted DNA from the expanded clones and genotyped an average of 110 polyG loci per clone and identified somatic mutations that either shortened or lengthened the polyG tract (genotyping data for mouse 1 and 2 shown in Additional file 2: Table S2 and Additional file 3: Table S3, respectively). Figure 1a shows how many different mutant alleles are identified for each marker across all of the approximately 100 cells genotyped for each mouse. Combining data from all cells harvested, each mouse exhibits an average of 0.5 mutant alleles/polyG locus/cell, which is more than one hundred-fold greater than we previously observed (0.003 mutations/locus/cell) using mice with intact DNA repair machinery [14]. Figure 1b shows the number of polyG marker mutations detected per cell for each mouse (from among all approximately 110 markers). On average, for each cell, more than one third of the 110 polyG markers (mouse 1: 36.7%, mouse 2: 34.4%) exhibited a somatic mutation. It is worth noting that the SV40 T-antigen originates from a strain (mixture of CBA/Ca and C57BL/10) different from the one (C57BL/6J) than it is crossed into and that contains the MLH1 and DNA polymerase delta deficient alleles. Littermates therefore carry differing amounts of strain-specific DNA from each parent, most likely including at loci encoding other DNA fidelity factors as well as polyG markers. The similarity in mutation profiles between the two individuals suggests that the genetic effects induced by the deficiency in polymerase proof-reading domain and mismatch repair genes are unlikely to be influenced by differences between mouse strains.

We next experimentally assayed the mutation frequency at polyG loci. From each mouse we selected one muscle fibroblast and one preadipocyte cell line and isolated 12 single cells that were each passaged for a defined number (20) of doublings. For each of the 48 subclones, we genotyped 110 polyG loci and identified mutations that were not found in the parental cell line from which the subclones were derived. We calculate that mouse 1 muscle fibroblasts and preadipocytes exhibit equal mutation rates, with a mean of 0.010 mutations/division/polyG locus, while mouse 2 displays similar values (p=0.248), with an average of 0.012 and 0.013 mutations/division/locus for muscle fibroblasts and preadipocytes, respectively (Additional file 4: Table S4, with the genotyping data from which it is derived shown in Additional file 5: Table S5). These results indicate that mutation rates do not vary with cell type or between individuals and support the notion that mutations can be used as a “molecular clock” [19] to unbiasedly infer cell lineage histories in different tissues from different mice.

### Quantifying mitotic history of tissues

Cells within the body all originate from the zygote. We approximated the genotype of the zygote as being the most commonly observed allele for each locus, across all of the cells examined. Because mutations arise with regular frequency during mitosis, a measure of the genetic distance separating individual cells from the zygote is expected to be proportional to the number of mitoses those cells have undergone since conception [19]. We calculated genetic distance for tissues based on the mean number of pairwise allelic differences for the polyG markers, adjusting for missing data (data for mouse 1 and 2 in Additional file 6: Table S6 and Additional file 7: Table S7, respectively). Measuring this distance from the zygote for cells in each mouse suggests that fibroblasts from hindlimb muscle and preadipocytes from abdominal fat have undergone a similar number of mitoses, yet it is significantly fewer than those of vascular endothelial cells derived from the brain (Figure 1c). One potential explanation for this observation is that it simply takes fewer cell divisions from the point at which muscle and fat differentiation begins until their development is complete, compared to what is required for the formation of blood vessels in the brain. Alternatively, it is possible that these tissues all arise at a similar point during development, but that muscle and fat originate from a larger group of progenitor cells than vascular endothelium. In the latter scenario, endothelial cells of blood vessels would require relatively more cell divisions before committing to specified lineages in order to produce the large numbers of cells required during the tissue maturation process.

To distinguish between these two possibilities, we compared the pairwise genetic distance among single cell clones within each tissue as well as to the zygote. For the progenitor cell pool which gives rise to any tissue, this comparison yields an estimation of the relative number of mitoses which cells have undergone prior to tissue commitment, in contrast to how many mitoses those cells have undergone after commitment. For muscle and fat the distance between cells within each tissue is greater than their distance to the zygote (Table 1). In contrast, vascular endothelial cells demonstrate that they are about as distant from each other as they are to the zygote. Since we isolated a similar number of cells from those tissues (Additional file 1: Table S1), we minimized possible bias introduced by unequal sampling. The pattern observed in muscle fibroblasts and preadipocytes may be interpreted as showing that during organogenesis, these cells form a population of mixed lineages bearing various genotypes, instead of from a few closely related progenitors. Following organogenesis, mutations continue to accumulate in descendant cells derived from the mixed founder population, with the result that cells within an organ are more dissimilar to each other than they are to the zygote. Contrastingly, for brain vascular endothelial cells, organogenesis appears to initiate from a limited number of progenitors, and cells within the tissue appear to undergo a large number of cell divisions in order to fully commit to the specific lineage. In this case, the genetic distance of cells from the zygote is much greater and is comparable to the average distance of single cells within the same type of tissue.

**Table 1:** **Average genetic distance and the sample error of mean** (**SEM**) **for comparisons among single cell clones grouped by their tissue origins**

		**Mouse 1**		**Mouse 2**	
		**Average distance**	**SEM**	**Average distance**	**SEM**
**Left fibroblasts**	Intra‐tissue	0.289	0.003	0.334	0.009
	Inter‐tissue	0.316	0.001	0.349	0.002
	To zygote	0.213	0.007	0.260	0.010
	Left to right	0.304	0.006	0.332	0.007
**Right fibroblasts**	Intra‐tissue	0.315	0.004	0.305	0.005
	Inter‐tissue	0.324	0.001	0.334	0.002
	To Zygote	0.238	0.008	0.237	0.007
**Left preadipocytes**	Intra‐tissue	0.282	0.016	0.360	0.010
	Inter‐tissue	0.313	0.002	0.358	0.002
	To zygote	0.251	0.010	0.271	0.0019
	Left to right	0.293	0.003	0.336	0.011
**Right preadipocytes**	Intra‐tissue	0.287	0.008	0.292	0.008
	Inter‐tissue	0.307	0.002	0.330	0.002
	To zygote	0.235	0.015	0.225	0.014
**Left vascular endothelial**	Intra‐tissue	0.285	0.007	0.334	0.008
	Inter‐tissue	0.329	0.002	0.365	0.002
	To zygote	0.343	0.0111	0.333	0.014
	Left to right	0.317	0.011	0.0348	0.015
**Right vascular endothelial**	Intra‐tissue	0.331	0.007	0.321	0.008
	Inter‐tissue	0.339	0.002	0.354	0.002
	To zygote	0.342	0.011	0.316	0.015

Notably, in both mice, we observed that relationships are in general much closer for cells in the same type of tissue than they are for cells in different types of tissue (Table 1). An interpretation of this observation is that the fate of progenitor cells are specified early in embryogenesis and remain committed during the remainder of development. It appears that cell migration between different primordial tissues is rare; otherwise, genetic distances within tissues would be similar to those between different types of tissues.

This notion also applies when examining the relatedness of left-sided tissues to their right-sided counterparts (Table 1). Interestingly, we found that the distance between contralateral tissues of the same type is generally larger than it is for the distance between the same types of ipsilateral tissues; however, the genetic distance for contralateral tissues of the same type is still smaller than the average distance between unrelated types of tissues. This finding suggests that establishment of left and right polarity takes place after specification of lineages to individual tissues, and, subsequently, cells largely develop constrained to either side.

### Reconstruction of lineage relationships by distance-based methods

We next evaluated whether genetic distance information could be used to infer lineage relationships between tissues. We used two approaches (one based on the eBURST algorithm and another utilizing network analysis) for deriving clonal relationships between tissues and cells from genetic distance calculations.

**Figure 2:**
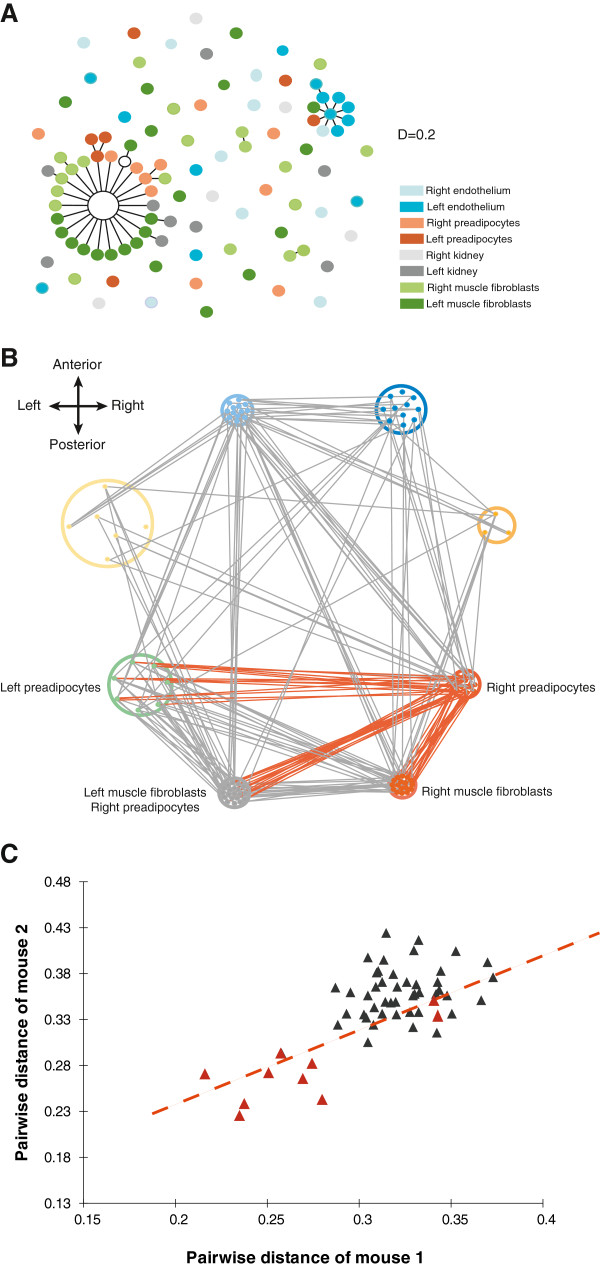
**Lineage relationships inferred from methods based on genetic distance.** (**A**) Modified eBURST analysis, showing “population snapshot” of single cell clones in mouse 1. Clusters of related single cell clones and individual unlinked clones are displayed as a single modified eBURST diagram by using the distance value D=0.2 as cut-off. Clusters of linked single cell clones correspond to complexes that share highly similar mutational profiles. Each single cell clone is represented as a dot with color indicative of its tissue origin. (Mouse 2 shown in Figure S1.) (**B**) Network representation depicting mutational similarities among single cell clones between both mice. Significant similarities between single cell clones for mouse 1 are shown with grey connecting lines. Each single cell clone is depicted as a dot with different colors indicative of tissue origin while the layout on the graph reflects relative anatomical location on the anteroposterior axis. The diameter of the circles correlates with the average distance within tissues. Orange lines show relationships that are conserved in mouse 2. (**C**) Scatter plot of distance between equivalent pairs of tissue, comparing mouse 1 to mouse 2. Distances of specific tissues to the zygote are colored orange; a trend line indicates their correlation. Among these comparisons, the distances between individual tissues to the zygote are largely conserved between the two mice.

We first adapted the eBURST algorithm [26], which was originally developed to display clonal relationships among bacterial populations. An advantage of eBURST analysis is that it may more sensitively detect clonal relationships in cases where there is insufficient genetic information to establish phylogeny. However, the algorithm is designed to interpret genotypes arising in haploid genomes. An additional limitation is its inability to analyze datasets as large as those generated in our study. To avoid these problems, our modified eBURST algorithm calculates relative genetic distances from pairwise comparisons of genotypes, connects isolates with related genotypes into groups and clonal complexes, and identifies the founding genotype of each clonal complex. Analysis using the modified eBURST algorithm suggests that muscle fibroblasts and fat preadipocytes are clonally related (mouse 1 shown in Figure 2a, mouse 2 in Additional file 8: Figure S1), in agreement with the above findings indicating that muscle fibroblasts and preadipocytes share a common population of progenitor cells. Only under such circumstances, is it possible for descendants of closely related lineages to localize and develop in physically separated tissues. For most clones, modified eBURST analysis does not detect meaningful relationships between other cell types. Nevertheless, given the fact that we examined only a small proportion of the cells present in any tissue, we are largely limited to detecting relationships between cells that are only separated by a few cell divisions. (Based on assumptions described in the Materials and Methods section, we estimate that the modified eBURST algorithm is limited to detecting clonal relationships of cells separated by ≤12 mitoses.) Intriguingly, modified eBURST analysis revealed in both mice several connections between single cell clones derived from distant tissues (such as from contralateral tissues), suggesting that cell migration may occur during development, such that spatially separated cells share similar mutation profiles. Overall, however, most cells from spatially isolated tissues did not exhibit such a relationship, suggesting that cell migration appears to be rare during development, at least across the physical distances separating cells within the tissues we sampled.

We then examined for similarities among cells through use of network analysis (Figure 2b), which offers a complementary approach for identifying ancestral relationships based on genetic distance [27]. In mouse 1, muscle fibroblasts and preadipocytes are most genetically similar, consistent with the findings reported above. The same close relationship between fibroblasts and preadipocytes appears in mouse 2, at least on the right side of the body; however, not all relationships in mouse 1 are preserved in mouse 2. To compare the overall similarity of tissue relationships between the two mice, we measured distances between the same pairs of tissues in both mice and calculated Pearson correlation coefficients (Figure 2c, based on data in Additional file 9: Table S8). This analysis demonstrates that the relatedness of different tissues to the zygote is largely the same in both mice (Pearson correlation coefficient=0.789, R^2^=0.622, and p=0.0067), but the relatedness between any two different tissues in the pair of mice follows no discernible pattern (Additional file 10: Table S9). We reconcile these observations by proposing that in different individuals, tissues develop at similar times with similar sizes of progenitor cell populations, but that the genetic composition of those progenitor cells is randomly assigned. Although the overall coefficient index for all pairs of tissues demonstrates that tissue relationships between these individuals are far from perfectly correlated, it is nonetheless non-random; in other words, the overall pattern represented in two mouse littermates reflects a combination of deterministic and stochastic developmental events.

### Phylogenetic reconstruction of lineage relationships

**Figure 3:**
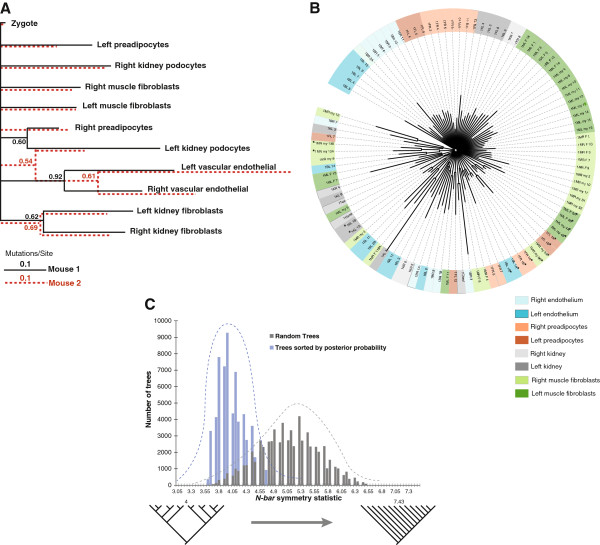
**Phylogenetic reconstruction of tissues and single cell clones in both mice.** (**A**) Phylogenetic tree of tissues, with mouse 1 in black and mouse 2 in orange, overlaid. Numbers at bifurcations indicate Bayesian posterior probabilities. (**B**) Phylogenetic tree of single cell clones in mouse 1. Only branch structures with larger than 50% posterior probability are shown. Pairs of single cell clones from the same parental cell are marked with asterisks. (Mouse 2 shown in Additional file 8: Figure S2) (**C**) Distribution of *N-bar* symmetry statistic for mouse 1 tissue trees with highest posterior probabilities compared to random trees with the same number of branches, showing a symmetric nature of the actual tissue trees. Examples of symmetric (left, *N-bar* = 4) and asymmetric trees (right, *N-bar* = 7.43) are shown. (Equivalent distribution for the *I*_*c*_ symmetry statistic are shown in Additional file 8: Figure S3).

In order to more specifically infer lineage relationships among cells from each mouse, we used the genotypes of individual cells, as well as collectively the mean genotype across tissues, to infer phylogenetic trees. We first computed genotypes for particular tissues based on the most frequent alleles found in all cells from the same type of tissue. Phylogenetic reconstruction of the different tissues (Figure 3a) demonstrates that, among all the types of tissue investigated in this study, vascular endothelial cells from the left and right sides of the brain share the most recent common progenitor and are therefore most closely related (as was found above in analyses based on mitotic distances). Fibroblasts from the left and right kidney are also closely related. Notably, these relationships are conserved in both individual littermates. Other tissues demonstrate more variable relationships, however. In comparing the two mice, for instance, kidney podocytes are more similar to preadipocytes in mouse 1 while they are closest to vascular endothelial cells in mouse 2. Despite the findings from the distance-based analysis, we failed to discern any relationship between preadipocytes and muscle fibroblasts using phylogenetic inference. This may be a consequence of using the zygote genotype as an outgroup in the phylogenetic reconstruction. It is possible that the similarity of genotypes of muscle, preadipocytes and zygote, demonstrated by distance-based clustering and network analyses, pose difficulties in resolving those groups from one another when employing phylogenetic analysis and, consequently, does not produce an informative tree structure.

When applying phylogenetic analysis to individual cells (as opposed to the composite genotype produced from cells of the same tissue type, as shown in Figure 3a), the number of somatic mutations identified was insufficient to produce well-supported bifurcating trees through phylogenetic reconstruction (mouse 1 shown in Figure 3b and mouse 2 in Additional file 8: Figure S2); half of terminal branches cannot be fully resolved and appear as polytomies. Employing even a low threshold of 50% Bayesian posterior probability yielded a tree in which all branches correspond to terminal bifurcations of pairs of cells, without revealing complex internal branching structures. Although this topology is limiting, there are nevertheless several noteworthy findings contained in the phylogeny. First, internal control clones that were split from the same parental clone in culture are largely paired together with high confidence (mouse 1: 16/18 paired with an average of 0.99 posterior probability; mouse 2: 26/28 paired with an average of 0.97 posterior probability), indicating neither that mutations occurring during *ex vivo* expansion nor that errors in determining marker genotypes are of sufficient magnitude to influence phylogenetic reconstructions. Second, pairs of single cell clones from different tissue origins occur frequently (mouse 1: 9/14; mouse 2: 8/11). Compared to pairs of phylogenetically related cells derived from the same tissue, pairs of phylogenetically related cells from dissimilar types of tissues exhibit longer branches connecting them to their most recent common progenitor. This finding indicates that such cell pairs diverge from their common ancestors substantially earlier in development than for related cells from the same tissue, confirming observations from our earlier studies [14]. Reassuringly, phylogenetically related pairs of cells from different tissues also had higher degrees of genetic similarity in our distance-based analyses and similarly formed statistically significant connections in the modified eBURST and network analyses. Altogether, the paired patterns of single cell clones in the phylogenetic reconstruction are consistent with cell mixing and migration occurring during embryogenesis. Yet, cell mixing and migration appear restricted to certain developmental stages and/or certain types of tissue, because, by and large, cells develop in a constrained space that is likely defined by interactions with neighboring cells and surrounding tissue architecture.

### Patterns of cell growth inferred from the shape of the tree

The topology of a phylogenetic tree is shaped by the process through which it has grown [28,29]. For example, if a lineage bifurcates, but only one of the subsequent two cell lines persists, then the shape of the tree will be asymmetric at that branch. For a tree produced from composite genotypes representing cells of the same tissue type (as in Figure 3a), these properties translate to the probability that progenitor cells will give rise to distinct tissue types. We therefore examined the topology of phylogenetic reconstructions for nonrandom shapes. We first generated a comparison set of trees based on randomization of genotypes. Assuming the same total amount of genetic information, we generated random genotypes with the same number of samples from our experimentally observed genotypes by sorting alleles of each locus into arbitrary orders. We used Bayesian phylogenetic analysis, collected the 5×10^4^ highest-scored trees and measured their degree of asymmetry. The results are shown in the histogram in Figure 3c, in which asymmetry is measured by the *N-bar* statistic [30]. (We also measured asymmetry using a different statistic, Colless’ imbalance statistic *I*_*c*_[31], which produced similar results, Figure S3.) Although the trees shown in Figure 3a are symmetric, they correspond to a Bayesian consensus estimating the single best tree. To get a sense of the range of the shapes of trees that are compatible with the experimental data for mouse 1, we collected the 5×10^4^ highest-scored trees (of 2.5×10^5^ total) produced by the phylogenetic analysis, measured their asymmetry, and superimposed the result on the values for the trees generated from random genotypes (Figure 3c, which shows symmetry measured by *N-bar*, and Figure S3, which shows symmetry measured by *I*_*c*_). Compared to trees based on randomized genotypes, possible phylogenies best fitting the experimental data are much more symmetric. We reject a trivial explanation that the symmetry arises from polytomies, where the branching order cannot be resolved, because the posterior probabilities support the inferred structures. The most obvious biological explanation for a symmetric tree is that there is no variation in speciation and/or extinction rates for different branches of the tree. With respect to embryogenesis, this implies that distinct types of tissue, represented by individual clades in the phylogenetic tree, each have a similar probability of descending directly from the zygote, at the root of the tree. Overall, this observation suggests that a population of pluripotent cells in the early embryo contributes to different lineages without bias and that the determination of lineage commitment during development is itself a stochastic event.

## Discussion

In our previous studies [13-16] employing phylogenetic analysis of somatic mutations accumulating during development, we analyzed only individual mice. Additionally, we had previously not taken advantage of genetic strains in which there is reduced DNA replication fidelity with correspondingly higher rates of somatic mutation. In the results we present here, comparison of tissue relationships in two sibling mice with mutator phenotypes reveals details about how well overall patterns of development are conserved between different individuals. Results from our distance-based analysis point to a stochastic model of development, in which progenitors of different tissues and their exact genetic composition are randomly determined. Additionally, the highly symmetric shape of reconstructed cell lineage trees in these mice, generated by phylogenetic inference using mutations accumulating in single cells, similarly supports the apparently stochastic nature of lineage differentiation occurring during embryogenesis.

Ever since Waddington first proposed a probabilistic model for how gene regulation modulates development in 1957 [32], stochastic contributions to cell fate determination have been repeatedly demonstrated in studies employing various linage tracing techniques, including dye injection [33], retroviral marking [34], and chimeras formed from embryonic stem (ES) cells obtained from mixtures of differently pigmented mouse strains [35]. For example, with respect to the latter, sibling littermates exhibit variable patterns of pigmentation, indicating that, at least in skin, mature tissues are randomly derived from primordial progenitors. Yet, the simple fact that most mice (and other individuals within a species) are patterned more-or-less the same suggests that there are limits to stochastic effects occurring during differentiation. A goal of our study was to determine where and when such restrictions might occur.

Developmental stochasticity has been mathematically modeled and experimentally concluded to be an inescapable consequence of gene transcription [36,37], epigenetic gene regulation [38] and protein interaction [39]. Ultimately, these processes presumably reflect the inherent noise in the networks into which genes and their products assemble, as governed by statistical and quantum mechanics [40-42]. However, this is not to say that development is solely a random process, as our data also indicate that during lineage specification, the timing and numbers of progenitor cell populations appear to be conserved between individuals.

An immediate question is how and why certain developmental events occur predictably while others appear to be random. Although our study does not provide direct clues, it is reasonable to speculate that such a balance between stochasticity and determinism is an evolutionary consequence that defines one species and distinguishes it from another but that at the same time allows for beneficial diversity within a species, promoting survival of at least some individuals in the face of a continually changing environment. This interpretation is somewhat analogous to the concept of genetic “buffering,” in which populations may tolerate otherwise deleterious mutations in genes in order to maintain higher genetic diversity and thereby expedite the rate of adaption [43]. Overall, our study offers genetic evidence to separate variable developmental events from conserved ones, and delineates a model in which development represents the sum of what can be efficiently specified in the genome balanced against the effort required to control entropic noise intrinsic to the underlying biochemistry.

**Figure 4:**
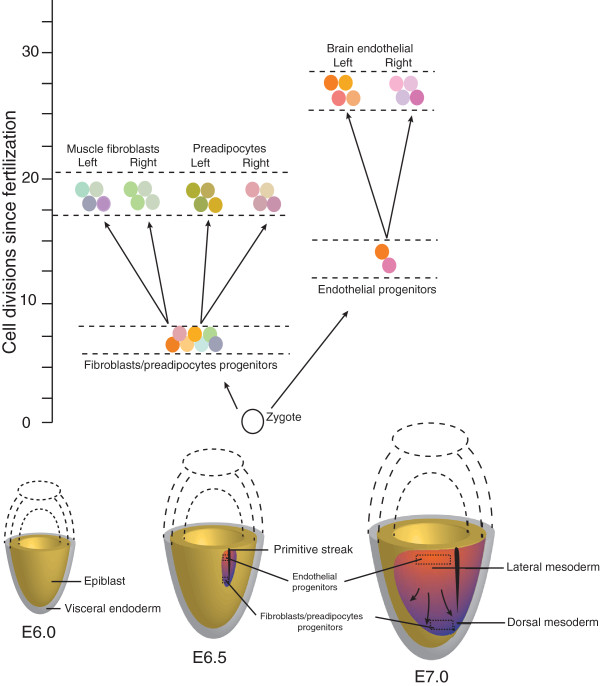
**Developmental model.** As gastrulation begins at around E6.5, the primitive streak forms and extends through the midline, establishing the anteroposterior body axis. During the process, mesoderm ingresses and begins to migrate to its ultimate position, where it will give rise to fibroblasts in muscle, preadipocytes, and endothelium. The progenitors of muscle fibroblasts and preadipocytes might arise earlier when mesoderm forms, starting from a pool of cells with fewer cell divisions (ranging from 6 to 8 divisions), while progenitors of brain endothelial cells could arise later from a few cells with a lengthier cell division history (ranging from 12 to 15 divisions). Once progenitors are established, those tissues may require similar numbers of further cell divisions to mature and develop into left and right compartments. The differing genetic identities and relative size of the progenitors for fibroblasts and preadipocytes are represented by differently colored spheres, and cell division history is indicated during mesoderm formation by color gradient, in which cells with fewer divisions appear more darkly colored. Numbers of cell divisions were calculated from the average genetic distance summarized in Table 1 using the mutation rate 0.010 for mouse 1 and 0.013 for mouse 2 as observed in this study. Schematic adapted from [46].

One of the most significant events during development is gastrulation, when the single-layered blastula reorganizes into the three classic germ layers, which subsequently give rise to specialized cell types. Given that muscle, fibroblasts, and fat share a common mesodermal origin, significant effort has focused on deciphering genetic mechanisms determining lineage commitment of progenitor cells to one cell type or the other [44,45]. However, the relative timing of lineage determination and the ultimate source of progenitors of muscle and fat are still unknown. In this study, by inferring from similarity in somatic mutations in individual single cell clones isolated from various tissues, we show that muscle fibroblasts and preadipocytes are similar in genetic composition we show that muscle fibroblasts and preadipocytes are similar in genetic composition and separate into discrete lineages at a similar time during development. Our data suggest that both of these tissues may descend from a pool of progenitor cells with mixed lineages, instead of from a single or a few progenitor cells with similar mitotic histories. We present a schematic, modeling these findings in Figure 4.

This notion resonates with recent discoveries of postnatal mesenchymal stem cells (MSCs), a type of cell that holds the potential to differentiate into multiple lineages in muscle, fat, and bone tissues, and which have been located as nonhematopoietic cells in bone marrow [47-49], pericytes encircling capillaries and microvessels [50], adipose tissue [51], and indeed from almost every postnatal connective tissue [52]. Given such a diversity of postnatal MSCs in various anatomical locations, it is reasonable to speculate that they could be derived from precursors with different genetic composition. We therefore propose a developmental model in which at the early three germ layer stage, there might be a large pool of progenitor cells within mesoderm that possess multiple lineage differentiation potentials, yet they themselves arise from proliferative growth and can be distinguished from each other by the mitotic mutations they bear. Such a mixed pool of progenitor cells gives rise to precursors that initiate formation of muscle, fat, and other cell types. While most of these progenitor cells differentiate and contribute to tissue formation, a few of them might persist as multipotent cells in these tissues postnatally through continuous self-renewal, providing a stem cell source for regeneration.

Another finding pertains to the establishment of lateral compartmentalization during mouse development. We conclude that the formation of tissue primordia is followed by the very early establishment of the left and right sagittal compartments within various tissues. Subsequently, cells mainly develop in their left or right territory with restricted cell migration in-between. Among individuals, such a development scheme could vary in terms of where exactly progenitor cells come from; however, the overall timing of lineage determination and the size of the founder population are largely conserved. At later stages of development, some tissues (for example, muscle and fat, as studied in our case) arise from a mixed pool of progenitor cells in the germ layer, while other tissues (for instance, vascular endothelium in brain, also as we have shown here), are derived from a single or at least limited population of progenitor cells. The phenomenon that we describe may become manifest in human disorders caused by somatic mutations with restricted laterality. For example, Proteus syndrome has been recently found to result from somatic mutations arising during embryonic development in *AKT1*[53]; a feature of Proteus syndrome can be hemihypertrophy [54], in which there is overgrowth of multiple tissues in a mosaic pattern but affecting only one side of the body, either right or left, with respect to the sagittal plane.

## Conclusions

Our studies initiate an investigation into differentiating between conserved and variable features of mammalian development. A considerable amount of experimentally-derived molecular genetic information (based on several hundred thousand PCR reactions) was required to generate the mutational data required for analysis here. Yet, yet, not all lineages are equally presented in our study due to their failure to survive in the clonal expansion, and the conclusions that can be drawn from studies based on just two simultaneously studied individuals are necessarily limited. Estimates of the degree of conservation of development from one individual to the next may be overestimated, as it possible that adding additional specimens would reveal a greater distribution of variable events. Nevertheless, given the extremely large number of possible lineage trees for the number of cells sampled in this study, however, it is improbable that the lineage similarities we have observed between a pair of mice have occurred by chance alone, and therefore the mere fact that lineage similarities were detectable at all in these studies is a necessarily meaningful finding. We look forward to technological advancements that will facilitate identification of mutations for the purposes of inferring cell lineage. Along those lines, we [16] and others [21] have recently demonstrated how deep sequencing holds promise in this regard. As cell fate maps become available for greater numbers of cells at increasingly higher resolution, and from multiple specimens of the same species, it should become easier to distinguish genetically determined variation from effects attributable to uncontrollable and random events occurring during embryogenesis. Such information could prove particularly valuable in sorting out birth defects where, for some, *de novo* single gene and chromosomal mutations are increasingly recognized as causative, yet for others, older concepts relating to disruptions of developmental events (without necessarily invoking genetic factors) still hold sway: a case in point being the “Robin Sequence”, in which multiple genetic and idiopathic factors contribute to human mandibular birth defects [55].

## Methods

### Mouse strains

Mouse studies were approved by the University of Washington Institutional Animal Care and Use Committee (Protocol 3015–04). *Pold1*^*+/e*^*Mlh1*^*+/Δ*^ mice were obtained from B. Preston (University of Washington) [16]. The DNA polymerase delta gene *Pold1* retained an inactive exonuclease domain due to a single point mutation (D400A) [23,24], while the mismatch repair gene *Mlh1* was dysfunctional due to the deletion of exon 2 [25]. In order to obtain desired cell replication capability *in vitro*, we employed the *H-2K*^*b*^-tsA58 transgenic mice (“Immortomouse”) strain, whose cells can be conditionally immortalized as driven by an interferon-inducible and temperature-sensitive form of the simian virus 40 large tumor antigen gene [22]. Homozygous *H-2K*^*b*^-tsA58 transgenic mice were separately bred to heterozygously deficient *Pold1*^*+/e*^*and Mlh1*^*+/Δ*^ mouse lines. The resulting lines were crossed to each other and were then mated amongst themselves to produce the mutant *Pold1*^*+/e*^*Mlh1*^*Δ /Δ*^*H-2K*^*b*^-tsA58^+/−^ mice used for our study.

### Cell isolation and culture

Kidney, abdominal fat tissue, muscles from the hindlimb, and brain were dissected separately from two 5 week-old female *Pold1*^*+/e*^*Mlh1*^*Δ /Δ*^*H-2K*^*b*^-tsA58^+/−^ mice. Whole tissues were minced and cells were separated by digestion with 0.5mM EDTA, 15 U/ml papain (Roche), and 200ug/ml Dnase I (Roche). To release cells from brain tissue slurries, samples were passed through Potter-Elvehjem tissue grinders. Fat and muscle from the same axial locations were subjected to vigorous pipetting. Kidney was broken down by filtering tissues through a 40-mesh screen. Cells were seeded into 15 cm culture dishes at dilutions yielding well-separated single cells, and clones arising from those single cells that survived were further isolated using cloning cylinders followed by deposition into single wells. Cells were cultured in DMEM/F12 media (Gibco/Invitrogen) containing 20% fetal bovine serum (Gibco/Invitrogen), 200 ng/ml mouse interferon gamma (R&D Systems), and penicillin G (100 U/ml) plus streptomycin (100 μg/ml) at 33°C with 5% CO_2_ and 5% O_2_ in a humidified incubator.

### Mutational analysis

Clones were expanded to approximately 10^6^ cells, and DNA was extracted using ArchivePure DNA Cell/Tissue Kit (5prime). 2 ng of DNA was used in each 5 μl PCR reaction consisting of 1 μM of oligonucleotide primers, 200 nM dNTPs, 0.05 U Taq DNA polymerase in 1× manufacturer-supplied buffer (Qiagen). For each primer pair, the forward primer was fluorescently tagged while the reverse primer was tailed with 5’-GTTTCTT-3’, as detailed in [14]. Primers used in the study are listed as in Additional file 11: Table S10. PCR products were diluted in 8 μl of Hi-Di Formamide (ABI/Life Technologies) with 0.02 μl GeneScan 500 ROX Size Standard (ABI/Life Technologies) per lane and subject to capillary electrophoresis on a 3730xl DNA Analyzer (ABI/Life Technologies). All reactions were carried out in 384-well plates, and liquid handling was performed on a Matrix Platemate 2×3 Pipetting Workstation (Thermo Scientific). Two of the 138 primer sets generated a second set of bands of unexpected size that could not be accounted for based on known genomic sequence. Nevertheless, these additional markers were reproducible and demonstrated variation independently from products corresponding to the expected marker sizes. We presume that they correspond to adventitious amplification of sequence unique to our strain or not compiled in the published mouse genome, and we included this information for analysis.

### Genotype interpretation

Results generated by the 3730xl DNA Analyzer were imported into GeneMaker 1.4 (Softgenetics) for automated fragment alignment and size calling. To minimize “stutter” artifacts from PCR amplification of repetitive sequences, independent triplicates of PCR amplification were performed for each single cell clones on each polyG loci, and manual size calling was further performed on each locus to ensure accuracy. Specifically, homozygous or heterozygous alleles that were consistent among the triplicates were defined based on three parameters: I_1H_, I_2H_ and I_3H_, corresponding to the fluorescent intensity (U) of the highest, second- and the third-highest signals, respectively. Homozygote genotypes were assigned when│(I_1H_-I_2H_)-(I_1H-_I_3H_)│ ≤ 10^4^ U (e.g. 106/106); heterozygote genotypes were assigned when│(I_1H_-I_2H_)-(I_1H-_I_3H_)│ ≥ 10^4^ U and I_2H_ (or I_3H_) > 0.8I_1H_ (e.g. 106/105), while signals with patterns falling in-between, or not reproducible among triplicates, were assigned ambiguously (marked as “X”, e.g. 106/X). Alleles were further assigned as being derived from one parent or the other so as to minimize the number of mutations required to generate the observed genotypes. Genotypes of zygote and individual tissues were defined as the most frequent alleles of all single cell clones as a whole or that of single cell clones from corresponding tissue types, respectively.

### Genetic distance calculation

In order both to handle missing data consistently and to allow for a diploid genome, we developed an algorithm for calculating genetic distance. Briefly, alleles of each pair of samples on each locus were compared and a distance was obtained by dividing the sum of minimal difference in length across all the loci by the number of loci examined. Loci that have more than one “X” (missing data) in a pair of single cell clones were not considered in the calculation. For pairwise comparison of tissues, all pairwise distances of single cell clones within compared tissues were averaged, and the significance was calculated by Student’s T-test against averaged distance of single cell clones of all tissues. The pairwise distances among single cell clones are further graphed in a network. Details of the algorithm are presented in Additional file 8: Supplementary Methods. The analysis was performed using a computer program (Additional file 12) written in the Python programming language.

### Modified eBURST clustering analysis

The eBURST algorithm has been employed to address clonal relationships of bacterial populations [56-59]. In our adaptation, an empirical threshold value was assigned, and only isolates having smaller distance were grouped clonally. The founding genotype was defined as the one that exhibited the smallest distances to the largest number of other members in the same group. In our modified eBURST algorithm, because markers were randomly selected from throughout the genome without respect to location within genes or other functional elements, mutations from different loci are weighed equally, and the relative distances of genotypes therefore represent the relatedness of the genotypes. A distance of 0.2 was used as the threshold, since this is equivalent to the distance of cells separated by 15 cell divisions, based on the observed mutation rate of 0.013 mutations/division/locus in the hypermutable mouse strain used in this study. (Distance value = mutation rate × number of cell divisions × number of loci genotyped, in this case, 0.2 = 0.013 × 15 × 1.) Our modified eBURST analysis was performed using a computer program (Additional file 12) written in the Python programming language.

### Phylogenetic reconstruction

Phylogenetic trees of cells isolated from the two mice were constructed using Bayesian inference as implemented in MrBayes 3.1 [60,61]. The standard data type was used and alleles on each locus were converted to a single digit from 0–9 according to their mutation patterns. A uniform distribution on the interval (0.05, 50) was used in the model of gamma-shaped rate variation across sites, and the parameter of the symmetric Dirichlet distribution was fixed to infinity. The Metropolis-coupled Markov Chain Monte Carlo method (MCMC) [62,63] was used to approximate the posterior probabilities of trees. MCMC samples from the first 5-6×10^7^ generations were discarded, and samples from subsequent 2-3×10^6^ generations were included for tree reconstruction.

### Measurement and statistical tests of the shape of phylogenetic trees

Randomized genotypes were generated by sorting genotypes in Additional file 3: Tables S3 and Additional file 4: Table S4 with arbitrary orders. Both random and experimentally observed genotypes were further used in Bayesian analysis as implemented in the MrBayes program to generate reconstructed phylogenetic trees with annotation of their posterior probability. Two measures that summarize the shape of a phylogenetic tree, *N-bar*[29] and Colless’ imbalance statistic *I*_*c*_[31], were calculated using the software package TreeStat (http://tree.bio.ed.ac.uk/software/treestat/). Distributions of *N-bar* or *I*_*c*_ values of reconstructed phylogenetic trees with the first 5×10^4^ highest posterior probabilities from both random and observed genotypes were overlaid with each other using graphing functions in Microsoft Excel.

## Competing interests

The authors declare that they have no competing interests.

## Authors’ contributions

WZ conceived and performed experiments, analyzed the data, and wrote the first draft. YT assisted with theory and writing software code. DJA performed breeding and assisted with obtaining mouse cell lines, interpreting data, and writing the manuscript. EMC performed genotyping and assisted in its interpretation. HR-B helped conceive experiments and design overall study. SJS helped conceive the study and assisted with cell line preparation and genotype performance and interpretation, as well as writing the final draft of the manuscript. MSH contributed to design and overall direction of the experiment and authored the final draft of the manuscript. All authors read and approved the final manuscript.

## Supplementary Material

Additional file 1 **Table S1.** Sources and numbers of cells isolated from each mouse. Click here for file

Additional file 2 **TableS2.** Genotype data for mouse 1, Microsoft Excel file. Click here for file

Additional file 3 **Table S3.** Genotype data for mouse 2, Microsoft Excel file. Click here for file

Additional file 4 **Table S4.** Mutation frequency among clonal isolates *in vitro*. Click here for file

Additional file 5 **Table S5.** Genotype data supporting Supplemental Table 5. Click here for file

Additional file 6 **Table S6.** Genetic distance data for mouse 1, Microsoft Excel file. Click here for file

Additional file 7 **Table S7.** Genetic distance data for mouse 2, Microsoft Excel file. Click here for file

Additional file 8 **Figure S1.** Modified eBURST analysis, showing “population snapshot” of single cell clones in Mouse 2. **Figure S2** Phylogenetic tree of single cell clones in mouse 2. **Figure S3** Distribution of *I*_*c*_ symmetry statistic for mouse 1 tissue trees with highest posterior probabilities compared to random trees. Click here for file

Additional file 9 **Table S8.** Pairwise genetic distance comparisons between mouse 1 and 2. Click here for file

Additional file 10 **Table S9.** Statistical analysis supporting tissue correlations in Supplemental Table 9. Click here for file

Additional file 11 **Table S10.** PCR primers for all PolyG markers used. Click here for file

Additional file 12 Software. Click here for file
