# Supplementary material for: Use of somatic mutations to quantify random contributions to mouse development
Source: BMC Genomics. 2013 Jan 18;14:39. doi: 10.1186/1471-2164-14-39 (PMC3564904; doi:10.1186/1471-2164-14-39)
Supplement: Additional file 8 — Figure S1. Modified eBURST analysis, showing “population snapshot” of single cell clones in Mouse 2. Figure S2 Phylogenetic tree of single cell clones in mouse 2. Figure S3 Distribution of Ic symmetry statistic for mouse 1 tissue trees with highest posterior probabilities compared to random trees. [file 1471-2164-14-39-S8.doc]

**SUPPORTING INFORMATION**

**
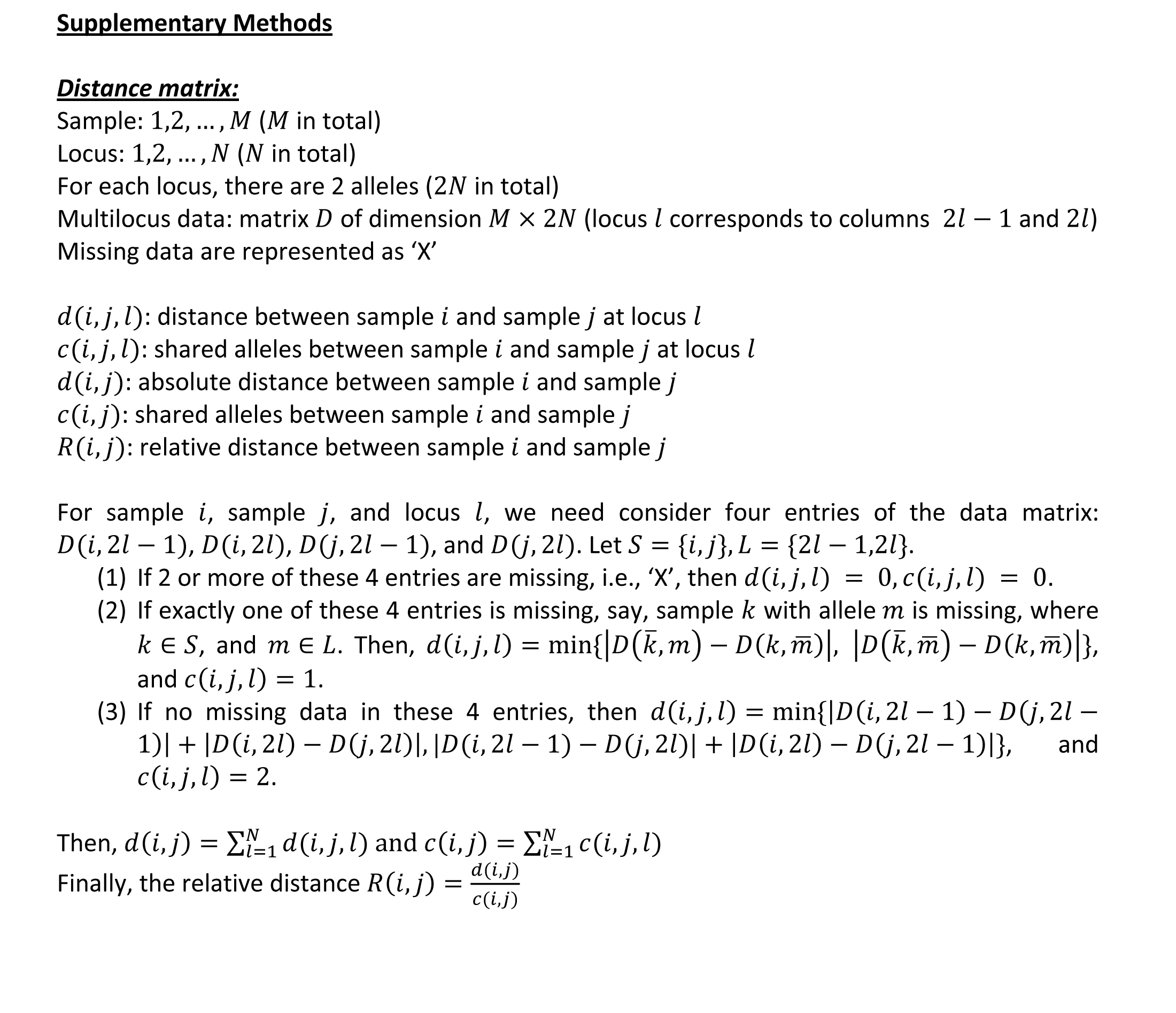
**

***
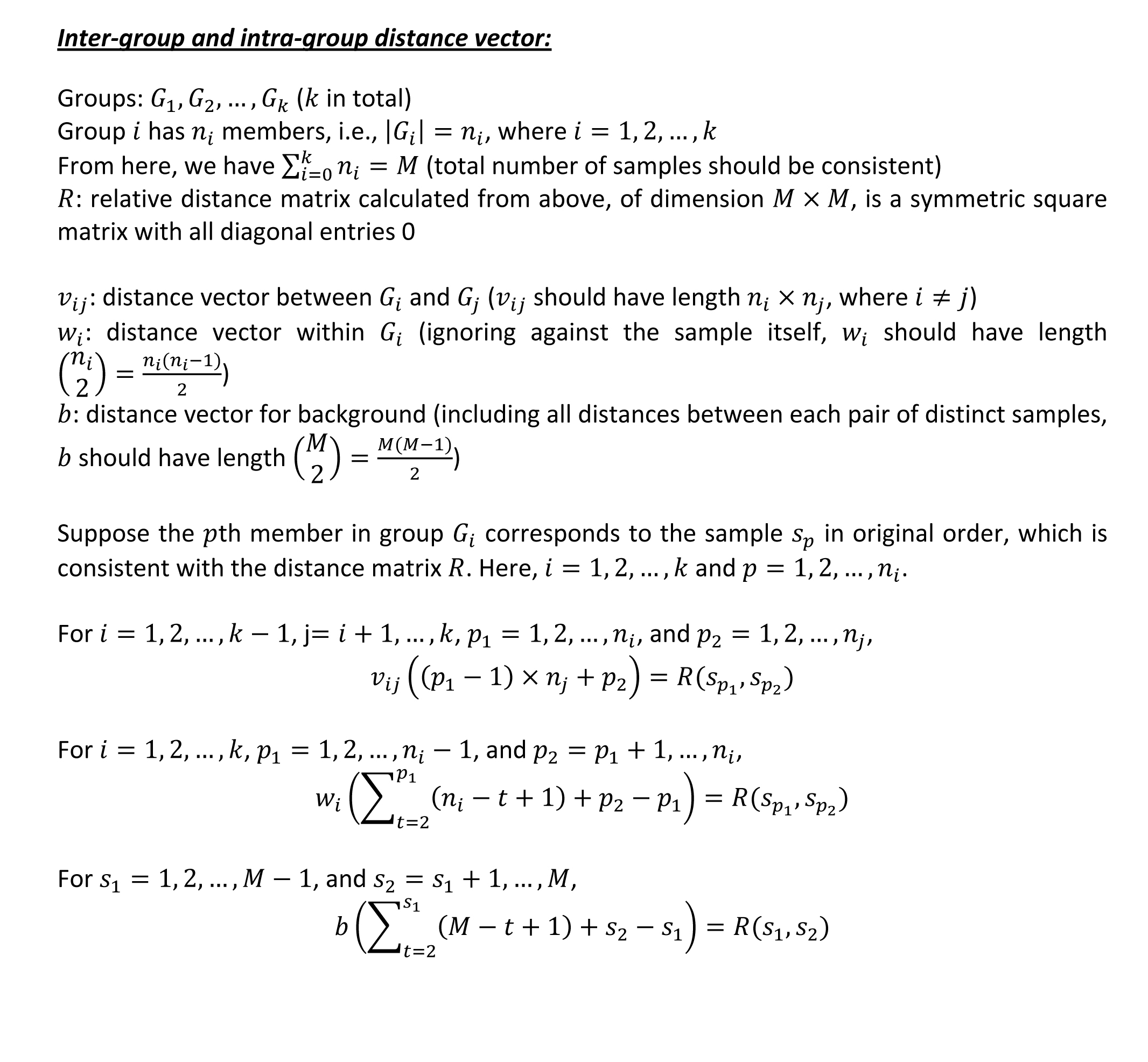
***

*
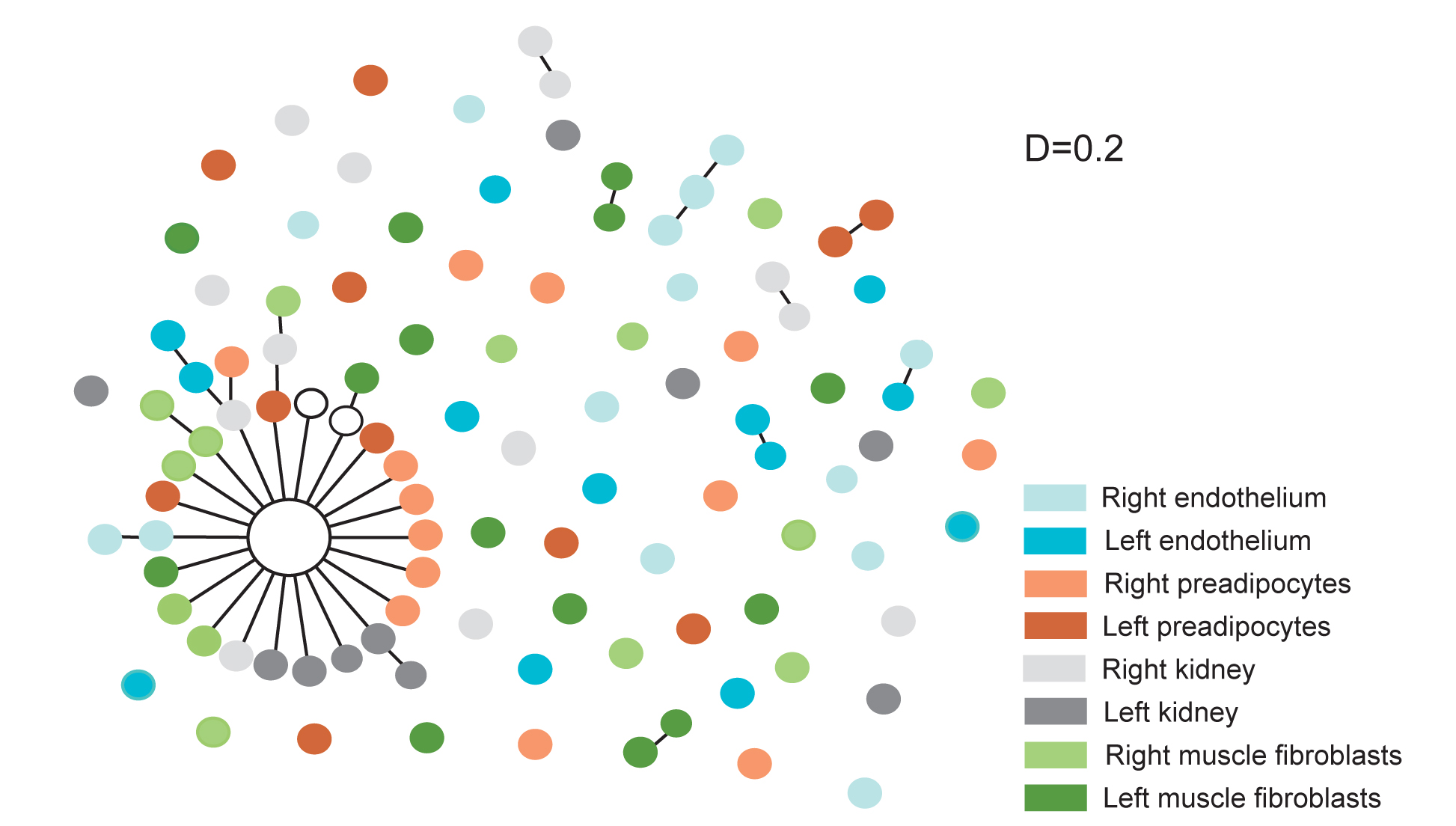
*

**Figure S1** Modified eBURST analysis, showing “population snapshot” of single cell clones in Mouse 2.

**
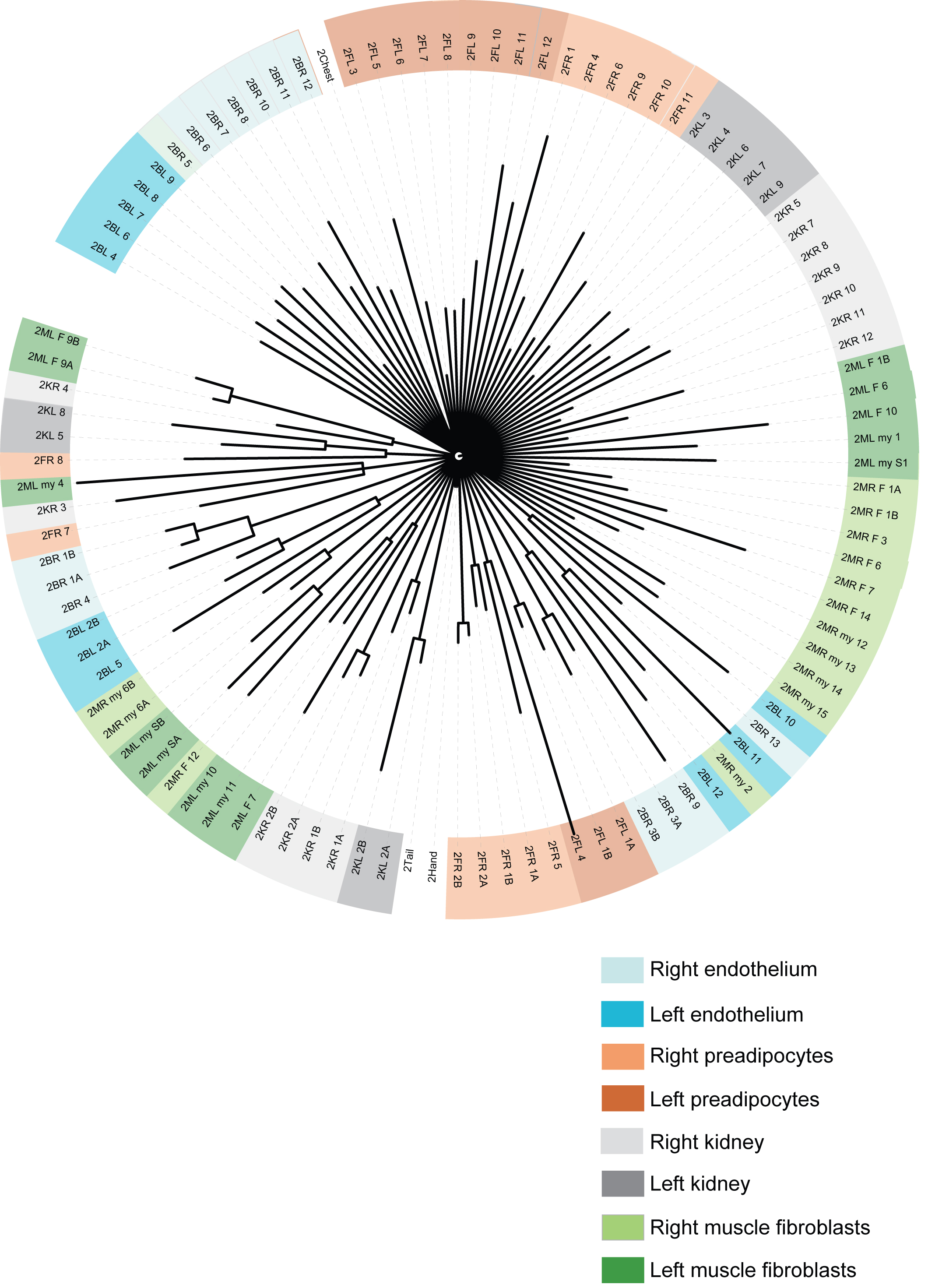
**

**Figure S2** Phylogenetic tree of single cell clones in mouse 2.


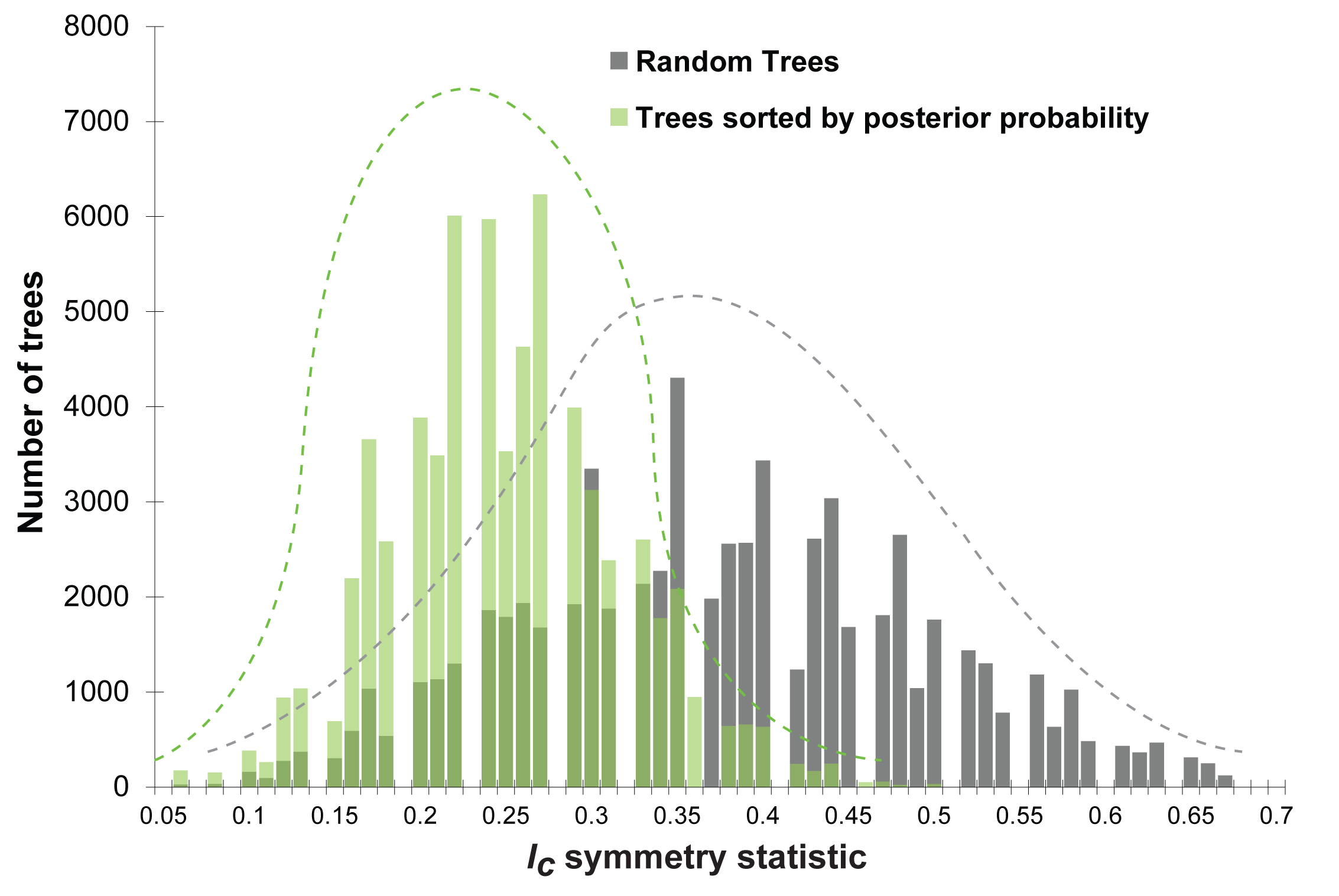


**Figure S3** Distribution of *I_c_* symmetry statistic for mouse 1 tissue trees with highest posterior probabilities compared to random trees.

Tables S1-S10 consist of large data archives in Microsoft Excel format and are contained in Zip-compressed file, data.zip.

**Table S1.** Sources and numbers of cells isolated from each mouse.

**Table S2.** Genotype data for mouse 1, Microsoft Excel file.

**Table S3.** Genotype data for mouse 2, Microsoft Excel file.

**Table S4.** Mutation frequency among clonal isolates *in vitro*.

**Table S5.** Genotype data supporting Supplemental Table 5.

**Table S6.** Genetic distance data for mouse 1, Microsoft Excel file.

**Table S7.** Genetic distance data for mouse 2, Microsoft Excel file.

**Table S8.** Pairwise genetic distance comparisons between mouse 1 and 2.

**Table S9.** Statistical analysis supporting tissue correlations in Supplemental Table 9.

**Table S10.** PCR primers for all PolyG markers used.

Software (program for calculation of genetic distance and the modified eBURST program) is contained in Zip-compressed file, software.zip.
